# Optical particle counter data collected in two inhabited sites close to an industrial hot spot during a three months survey

**DOI:** 10.1016/j.dib.2019.104250

**Published:** 2019-07-15

**Authors:** S. Licen, G. Barbieri, S. Cozzutto, M. Crosera, G. Adami, P. Barbieri

**Affiliations:** a Dept. of Chemical and Pharmaceutical Sciences, University of Trieste, Via L. Giorgieri 1, 34127 Trieste, Italy; b ARCO SolutionS s.r.l., Spin-off Company of the Dept. of Chemical and Pharmaceutical Sciences, University of Trieste, Via L. Giorgieri 1, 34127 Trieste, Italy

**Keywords:** Particulate matter, Ambient air, Optical particle counter, Statistical analysis, Steel plant

## Abstract

Data on this paper describe the monitoring of different size ranges of particulate matter on dwellings positioned close to an integral cycle steel plant. Data were collected by eight channel (PM_0.3_, PM_0.5_, PM_0.7_, PM_1_, PM_2_, PM_3_, PM_5_, PM_10_) optical particle counters positioned in two sites. The data were recorded as counts-per-minute for every size channel in a three months survey from June to September 2015. Basic statistical elaboration and boxplot graphs as well as raw data are included. The data are related to “Characterization of variability of air particulate matter size profiles recorded by Optical Particle Counters near a complex emissive source by use of Self-Organizing Map algorithm” Licen et al.,2019, in which a statistical elaboration by Self-Organizing Map algorithm is proposed.

**Table:** Specifications Table

Subject area	*Environmental Science (Pollution)*
More specific subject area	*Air quality monitoring*
Type of data	*Sampling site image, table, boxplot graphs*
How data was acquired	*Data were collected by two optical particle counters (OPCs – one positioned in each site) with eight channels (model 212 Eight Channel Particle Counter, Met One Instruments, Inc., Rowlett, Texas, USA). Size channel ranges: 0.3; >0.3-0.5; >0.5-0.7; >0.7-1.0; >1.0-2.0; >2.0-3.0; >3.0-5.0; >5.0-10.*0 μm*. The instruments continuously sampled air at* 1 l·min^-1^*and provided data count* per *minute for each channel.*
Data format	*Table is used to show basic statistics, boxplot graphs are used to show the comparison between the two sites, raw data are provided as well*
Experimental factors	*Counts-per-minute for each of the eight size channels were collected in two sites during a three months survey.*
Experimental features	*The counts-per-minute collected by each instrument were loaded in R software**[2]**as a text file and elaborated to obtain the basic statistics and the boxplot graphs*
Data source location	*The data were collected in Trieste, Italy*
Data accessibility	*Data are within this article.*
Related research article	*Licen, S., Cozzutto, S., Barbieri, G., Crosera, M., Adami, G., Barbieri, P.* "*Characterization of variability of air particulate matter size profiles recorded by optical particle counters near a complex emissive source by use of Self-Organizing Map algorithm" 2019 Chemometrics and Intelligent Laboratory Systems, 190, 48–54*[1]

**Table:** 

Value of the data • Variability of particulate matter size distribution (optical diameter 0,3–10 μm) at minute resolution during three months at two civil dwellings exposed to steel plant emissions is presented, allowing assessment of potential short term exposure to different type of air particulate matter; • The raw data included can be used to test different/new statistical models on an articulated real environmental data set (2 sites, 8 dimensional bins, about 100 000 minutes).

## 1 Data

**Fig. 1:**
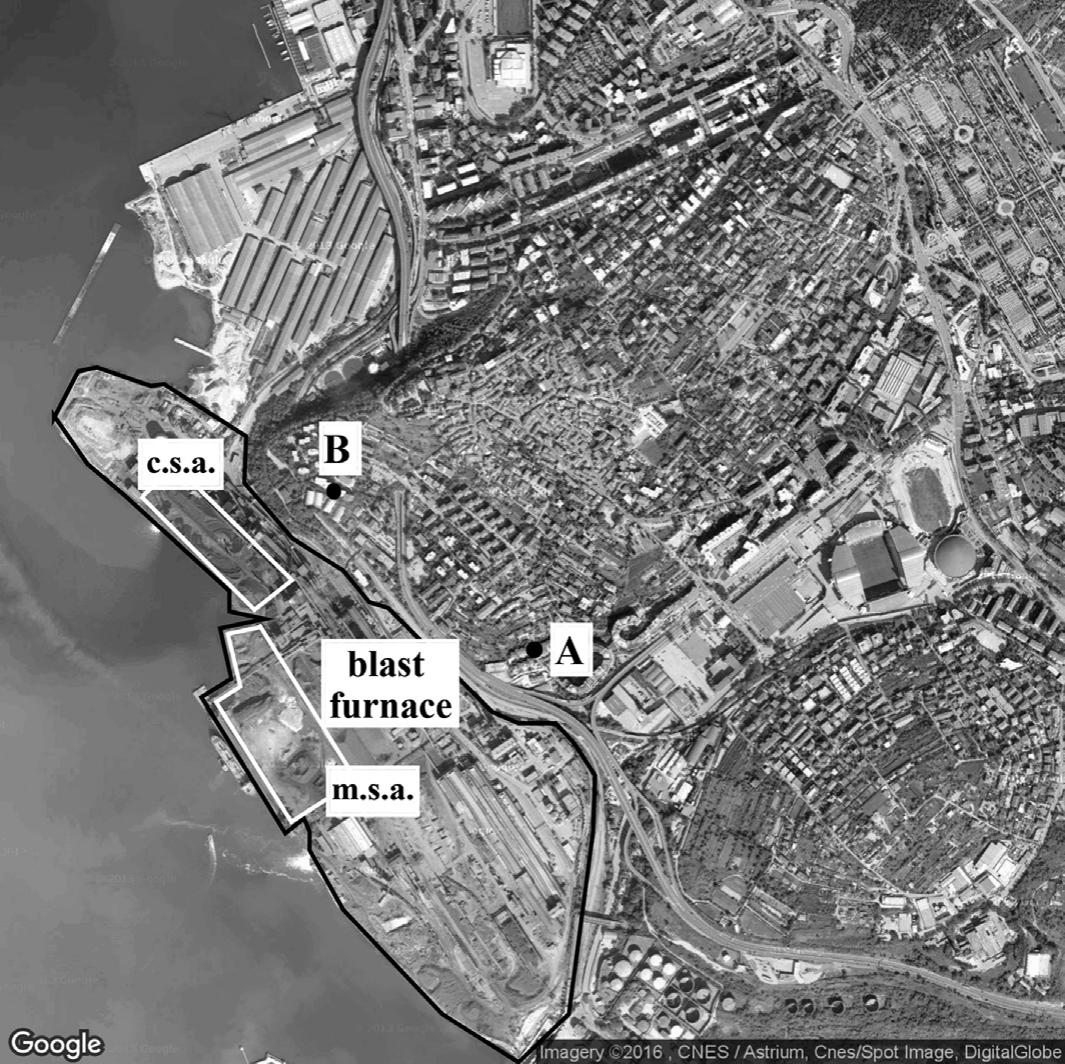
Map of the Trieste area which hosts the integrated steel plant. The sampling sites (A and B), the boundary of the steel plant (black line), the position of the blast furnace, the carbon storage area (c.s.a – white line) and the mineral storage area (m.s.a – white line) are highlighted.

**Fig. 2:**
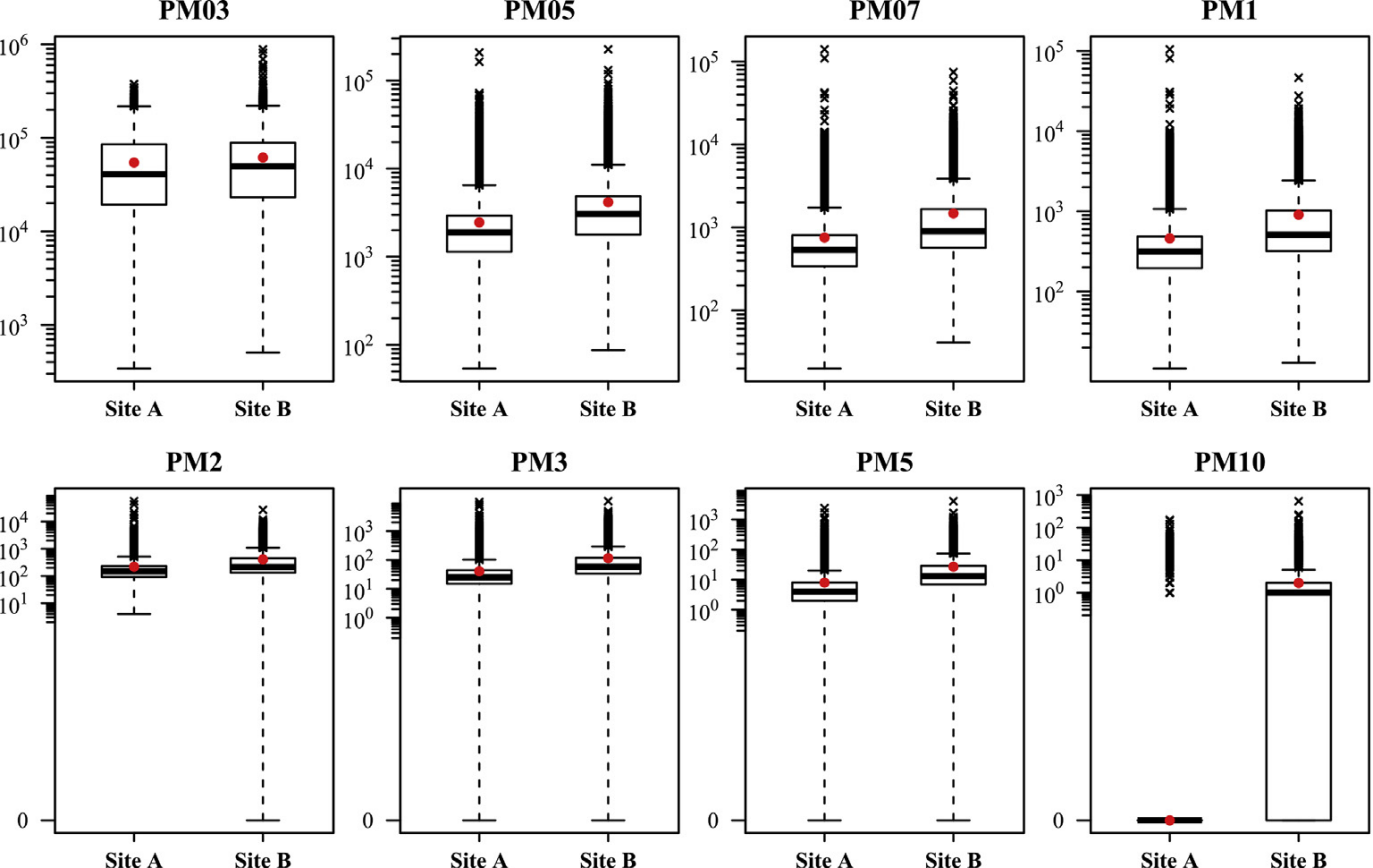
Boxplot graphs that compare the distribution of the data in the two sites for each PM size range (in counts per minute – y axis in log scale). The red dots represent the mean value. The cross shaped points represent the outliers.

The data presented describe monitoring of particulate matter (PM) at dwellings positioned near to an integral cycle steel plant. The counts-per-minute for 8 p.m. size ranges were collected in two sites (see map in Fig. 1) during a three months monitoring campaign (*5*th *June 2015 -10*th *September 2015*) by Optical Particle Counters (OPCs). Fig. 1 shows a map of the site where the data were collected. Table 1 displays a comparison of data collected in the two sites for each PM size range using basic statistics. Fig. 2 shows boxplot graphs to compare the distribution of the data in the two sites for each PM size range.

**Table 1:** Basic statistics for site A and site B (in counts-per-minute).

	Min		Median		Mean		Max	
	Site A	Site B	Site A	Site B	Site A	Site B	Site A	Site B
PM03	341	505	40816	49590	54592	61767	377209	885568
PM05	54	87	1892	3067	2459	4172	207524	225385
PM07	20	41	537	901	758	1483	140066	74506
PM1	11	13	317	511	462	911	104595	46388
PM2	4	0	148	214	220	411	57016	27345
PM3	0	0	25	58	41	116	10065	10766
PM5	0	0	4	13	8	27	2377	4058
PM10	0	0	0	1	0	2	174	651

## 2 Experimental design, materials, and methods

### 2.1 Site description

The data were collected in the city of Trieste (NE- Italy) near dwellings positioned close to an integrated steel plant. In the past years several studies were conducted on the site to assess different pollutant and odor impacts [3], [4], [5]. The main renown sources of particulate matter of the plant are the blast furnace [6], [7] and carbon and mineral storage areas. Site A and site B are within a radius of 350 m around the above mentioned sources.

### 2.2 Instrumentation and data collection

Data were collected by two optical particle counters with eight channels (model 212 Eight Channel Particle Counter, Met One Instruments, Inc., Rowlett, Texas, USA). The size channel ranges were 0.3; >0.3–0.5; >0.5–0.7; >0.7–1.0; >1.0–2.0; >2.0–3.0; >3.0–5.0; >5.0–10.0 μm. The channels will be named from now on in the text as PM03, PM05, PM07, PM1, PM2, PM3, PM5 and PM10 respectively. The instruments continuously sampled air at 1 l·min^-1^ and provided data count per minute for each channel. The three months monitoring campaign was conducted in the period from June to September (5th June 2015–10th September 2015) because it is characterized by the presence of sea breezes blowing from the sea to the inland, i.e., from the steel plant to the city.

### 2.3 Raw data

The dataset is presented in two comma delimited text files, one for each site. The filename identify the site. The header of the dataset reports the date/time of collection followed by the counts-per-minute for every channel size (see par. 2.2).

### 2.4 Basic statistics

The basic statistics for the data were evaluated in R software environment [2] and are reported in Table 1.

### 2.5 Boxplot graphs

The boxplot graphs were produced in R software environment [2] and are reported in Fig. 2.

A value has been considered as outlier if it was more than two times the interquartile range from the box.
